# Shortest Path Algorithm in Dynamic Restricted Area Based on Unidirectional Road Network Model

**DOI:** 10.3390/s21010203

**Published:** 2020-12-30

**Authors:** Haitao Wei, Shusheng Zhang, Xiaohui He

**Affiliations:** School of Earth Science and Technology, Zhengzhou University, No. 75 Daxue North Road, Erqi District, Zhengzhou 450052, China; zzu_wei@zzu.edu.cn (H.W.); zhangssgiser@gs.zzu.edu.cn (S.Z.)

**Keywords:** navigation, unidirectional road network, statistical parameter, path planning, restrict search area, Dijkstra algorithm

## Abstract

Accurate and fast path calculation is essential for applications such as vehicle navigation systems and transportation network routing. Although many shortest path algorithms for restricted search areas have been developed in the past ten years to speed up the efficiency of path query, the performance including the practicability still needs to be improved. To settle this problem, this paper proposes a new method of calculating statistical parameters based on a unidirectional road network model that is more in line with the real world and a path planning algorithm for dynamically restricted search areas that constructs virtual boundaries at a lower confidence level. We conducted a detailed experiment on the proposed algorithm with the real road network in Zhengzhou. As the experiment shows, compared with the existing algorithms, the proposed algorithm improves the search performance significantly in the condition of optimal path under the premise of ensuring the optimal path solution.

## 1. Introduction

With the rapid development and widespread application of technologies such as mobile geographic information systems (GIS), global positioning system (GPS), wireless sensor networks (WSN) and wireless communications [1,2,3,4,5,6], many people rely on the path provided by the navigation device to travel [7,8,9]. However, as the car ownership increases, the low capacity of urban roads limits the efficiency of traffic [10,11]. Therefore, how to choose an optimal driving route in the complex urban road network has risen lots of attention [12].

In network theory, this corresponds to the shortest path problem. The shortest path problem is to find the shortest directed path from a starting node to others in a directional network of arbitrary length which is one of the most basic problems in network optimization [13,14]. There are many algorithms for the shortest path including Dijkstra algorithm [15], A* algorithm [16], ant colony algorithm [17], genetic algorithm [18] and simu18lated annealing algorithm [19]. Dijkstra algorithm is the most commonly used algorithm to solve this problem on graphs with edge weights that are not negative. The main idea of Dijkstra algorithm is to spread circle by circle with the center, the starting node O, until the terminal node D is surrounded. Although the Dijkstra algorithm can effectively search for the shortest path between Origin–Destination (OD) pairs, it only considers the topological characteristics of the network in the design process. Dijkstra algorithm ignores the spatial distribution characteristics of the network so that the path search is not directional, so a large number of nodes that are not related to the shortest path are introduced into the calculation, which affects the efficiency of the algorithm. Therefore, higher resource cost and lower search efficiency limits the application of traditional Dijkstra algorithm in navigation software. Through the analysis of the optimization path based on the Dijkstra shortest path algorithm, it is found that the previous researchers mainly optimized the performance of the shortest path algorithm from four aspects: (1) optimization based on data storage structure [20]; (2) optimization based on network scale control [21,22]; (3) optimization based on search strategy [23,24,25,26]; and (4) optimization of priority queue structure [27,28,29,30]. This paper mainly optimizes Dijkstra algorithm from the control network scale.

Various optimization algorithms are developed to reduce the number of nodes because the time complexity of Dijkstra algorithm is proportional to the square of the number of nodes. Assuming that the number of nodes is proportional to the area of the search area, reducing the area of the search area is essential to this problem. In the transportation network, the direction of the route will be reflected if the starting point and ending point are determined. According to this network characteristic, Nordbeck [21] proposed a shortest path algorithm based on the ellipse, which limits the search node collection in a certain area and greatly narrows the search scale. Lu [31] proposed a shortest path algorithm with the minimum bounding rectangle of the ellipse as the restricted area. The experimental results showed that although the algorithm expanded the search area compared to the shortest path algorithm based on the ellipse restriction, there was no need for a large number of product and square root operations; therefore, the algorithm improved search efficiency. Fu [32] proposed that the line of the OD pair was set as the diagonal of the rectangle firstly, then, the threshold T_1_ was determined to expand the original rectangle and get the large one R, and finally the two sides of the diagonal of the rectangle R were set as the search area. Experimental results showed that compared with Dijkstra algorithm, the search scale of this algorithm was significantly reduced, which effectively improved the execution speed of the algorithm. Wang [22] proposed a shortest path algorithm based on a parallelogram to limit the search area. The algorithm used the smallest parallelogram containing an ellipse as the restricted search area. The experimental results showed that the search time required by the algorithm was less than the unlimited search in all cases areas and ellipses limit the search time required to search the area. Bu [33] proposed an improved algorithm that dynamically changed the search direction and restricted the search area in the dynamic search area based on the spatial characteristics of the road network. Compared with Dijkstra algorithm, this algorithm reduced the time and space complexity of the algorithm and improved the efficiency of the algorithm. Wang [34] proposed an optimal path algorithm that limited the search area based on the common characteristics of typical urban road networks. According to the different Euclidean distances of OD pairs, the algorithm searched for the shortest path in two types of ellipses of different sizes. Experimental results showed that when the Euclidean distance of the OD pair is far, the algorithm could reduce the time consumption by 33–47% compared with the ellipse restricted search area algorithm. Zhou [35] proposed a shortest path algorithm based on the corner direction rectangle. The algorithm was based on the direction rectangle and reduced the search area by cutting the corresponding corner area. The experimental results showed that the algorithm could significantly improve the search efficiency of the shortest path without affecting the reliability.

The various shortest path algorithms for restricted search areas proposed by the above scholars fully consider the characteristic attributes of the spatial distribution of the road network, including the positional relationship and distance relationship between the starting node and the ending node. The above algorithms all improve the efficiency of searching for the shortest path by narrowing the search area and reduce data redundancy. However, there are still some limitations, which can be summarized as follows:

1. Most of the road network data models used in the previous or current restricted search area algorithms are two-way road network models. However, the two-way road network model does not well represent the actual road conditions in the real world, and the statistical parameter results of the two road network models are quite different;
2. Most restricted search area algorithms are improved on the basis of the ellipse restricted search area algorithm. Therefore, a 95% confidence level is adopted in the process of calculating statistical parameters to ensure that most of the path solutions obtained are optimal. However, a higher confidence level has the problem of a larger search area;

In response to the above problems, this paper proposes unidirectional road network model (URNM) and shortest path algorithm based on virtual boundary in dynamic ellipse restricted area (SPAVBDERA). The data selected in this paper is the road network data of Zhengzhou, which includes highways, national highways, provincial highways, urban expressways and main urban roads in Zhengzhou. URNM was established on this basis. URNM regards the road as the basic element, uses the relationship between the roads, starts from the road network information and the path search efficiency, and establishes a road network model that meets the actual road needs. We have proved its practicality in the algorithm. Firstly, SPAVBDERA calculates different statistical parameters according to the Euclidean distance of different OD pairs, and selects a smaller confidence level to establish the algorithm search area, and then constructs a virtual boundary in the search area to ensure that the algorithm can obtain the complete solution of the optimal path. Finally, we compared the results of SPAVBDERA with Dijkstra algorithm and the shortest path algorithm for ellipse restricted search area.

The paper is organized as follows: In the Section 2, URNM and SPAVBDERA are elaborated. The Section 3 shows the experimental results of the proposed algorithm. The results of SPAVBDERA are further discussed in Section 4. The full text is summarized and the future research directions are prospected.

## 2. Algorithm Description

### 2.1. Unidirectional Road Network Model (URNM)

URNM is improved based on the graph structure model. The modeling idea of URNM regards the road as the basic element to build the model. URNM saves the attribute information and road traffic information of the road section on the road, which reduces data storage redundancy and improves the efficiency of querying the shortest path of the road network.

*G* = <*V*, *E*, *E_attr_*> (1)

In the Equation (1), *G* represents the road network; *V* = ($v_{0}$,...$,\;v_{n}$) represents the set of nodes in the network, $v_{i}$ is the intersection or endpoint of the road; *E* = <$v_{i}$, $v_{j}$> is the set of ordered vertex pairs, which indicates that there is one path from $v_{i}$ to $v_{j}$, $v_{i}$, $v_{j}$∈*V*, the direction of each path is a vector; $E_{attr}$ = <$E_{i}^{ID}$, $E_{i}^{LongS}$, $E_{i}^{LatiS}$, $E_{i}^{LongE}$, $E_{i}^{LatiE}$, $E_{i}^{Length}$, $E_{i}^{Direction}$> is a collection of path attributes, $E_{i}$ = < $v_{i}$, $v_{i+1}$>, $E_{i}$∈*E*, $E_{i}^{ID}$ represents the road to which the path belongs, and paths on the same road have the same ID; $E_{i}^{LongS}$ represents the geographic abscissa of the path starting point; $E_{i}^{LatiS}$ represents the geographic ordinate of the path starting point; $E_{i}^{LongE}$ represents the geographic abscissa of the end of the path; $E_{i}^{LatiE}$ represents the geographic ordinate of the end of the path; $E_{i}^{Length}$ represents the length of the path, where the path length discussed in this paper is a scalar, non-negative number, and all lengths are positive; $E_{i}^{Direction}$ is the path direction indicator, 1 means positive, 2 represents the reverse direction, and 3 represents the virtual boundary direction.

Compared with the ordinary plane network diagram, the road network diagram based on URNM usually has the following characteristics:

1. There are many points and edges, and the number of road sections connected to each node is mostly 2, 3, or 4, which is mostly a large-scale sparse network;
2. The network topology is complicated;
3. The side is one-way accessible;
4. There is a special reversing node in the network that indicates the vehicle turns around.

### 2.2. Dynamically Restricted Search Area Path Planning Algorithm

The classic Dijkstra [15] algorithm adopts a greedy strategy. This algorithm is a process of generating the shortest path in the order of increasing distance from the node to the starting node, which has great blindness and is always ready to go everywhere unfold. In this way, the search area finally swept is a circle with the starting point as the origin and the length of the line between the starting point and the terminal point as the radius. The time complexity of this algorithm is *O* ($n^{2}$), where *n* is the number of nodes in the road network. Therefore, the algorithm will generate a large number of redundant nodes in the process of traversing the nodes in the search area. This shortcoming promotes the research of related algorithms for reducing the search area. The traditional shortest path algorithm for restricted search area is proposed for the spatial distribution characteristics of road networks. Its purpose is to determine a small range in a large road network so that the shortest path between any given two nodes is most likely to be searched in this range. These algorithms improve the efficiency of searching for the shortest path and reduce data redundancy.

#### 2.2.1. Shortest Path Algorithm Based on Ellipse Restricted Search Area (SPAERSA)

SPAERSA is the shortest path optimization algorithm first proposed by [21] to narrow the search range. The algorithm defines an ellipse as a restricted search area, which means that nodes outside the ellipse are ignored during the search for the shortest path, thereby improving the efficiency of the shortest path search. The algorithm uses the starting point *O* and the terminal point *D* as the focus of the ellipse to construct the ellipse. The key to the ellipse limitation in the algorithm is to find the appropriate major axis *MD* of the ellipse. The length of *MD* is calculated by statistical analysis.

First, we systematically extract a certain number of representative nodes from the set of transportation network nodes, and construct two sets of nodes *G_O_* and *G_D_*. The Cartesian product *G_OD_* of these two sets can be given by Equation (2).

(2) $$G_{OD}\;=\;G_{O}\;\times \;G_{D}\;=\;\{(O,\;D)|(O\in G_{O})\;\wedge \;(D\in G_{D})\}$$

Each element in *G_OD_* can be regarded as the starting and terminal point of the shortest path to be found. Suppose the actual distance of a certain OD pair is $F_{OD}^{i}$, and the Euclidean distance is $E_{OD}^{i}$, so the ratio coefficient $R_{OD}^{i}$ = $F_{OD}^{i}/E_{OD}^{i}$ can be obtained. For sample, the ratio coefficient set *R_OD_* can be obtained. Then we perform statistical analysis on the elements in the *R_OD_* to obtain a specific value *f* so that the total number in the *R_OD_* is the elements that meet a certain confidence level, and this value is less than *f*. Finally, we use *f* as a constant multiplier and use the coordinates of the starting and terminal points to obtain the *MD* of the major axis of the ellipse. The specific process of the shortest path algorithm based on the ellipse restricted search area is shown in Algorithm 1. α is the Euclidean distance between two points, OPEN represents the temporary label set of nodes, and CLOSE represents the permanent label set of nodes. Γ represents the set of nodes in the restricted search area, CurrentNode represents the current node, AdjacentNode represents the adjacent node of CurrentNode, and S represents the shortest path.
**Algorithm 1** The Shortest Path Algorithm based on Ellipse Restricted Search Area *FindPathSPAERSA (G*,* O*,* D)***Input:**Graph *G*, origin O, destination D.**Output:**The shortest path from O to D.1:Set O and D as the focal points, α*_OD_* as the focal length, and *f* × α*_OD_* as *the major* axis length to establish an ellipse restricted search area;2:Put the node in the ellipse into Γ;3:*CurrentNode* ← O;4:Put *CurrentNode* into *CLOSE*;5:Put *AdjacentNode* into *OPEN* and *AdjacentNode* ∈ Γ;6:*CurrentNode* ← The adjacent node closest to *CurrentNode* in *OPEN*;7:**if** D = *CurrentNode*
**then**8:**return***S*;9:**else**10:Put *CurrentNode* into *CLOSE*;11 Put *AdjacentNode* into *OPEN* and *AdjacentNode* ∈ Γ;12:**if***OPEN* = **NULL then**13: Choose a larger *f* value, go to step 1;14:**else**15:Go to step 6;

The statistical parameter *f* calculated by SPAERSA is generally a constant multiplier that makes the total number of elements in the *R_OD_* meet the 95% confidence level. The algorithm takes into account the position relationship between the starting node and the terminal node and limits the search node set to an ellipse, which reduces the number of nodes that needs to be traversed during the search process, thereby achieving the goal of reducing time complexity. However, the higher confidence level makes the algorithm still need to traverse more nodes in the process of searching for the shortest path. Especially when the Euclidean distance of the OD pair is large, the algorithm will still generate a large number of redundant nodes, so the efficiency of the algorithm is sometimes even not as good as the omnidirectional search algorithm of unlimited search area.

#### 2.2.2. Fitting of Statistical Parameter Functions

**Figure 1:**
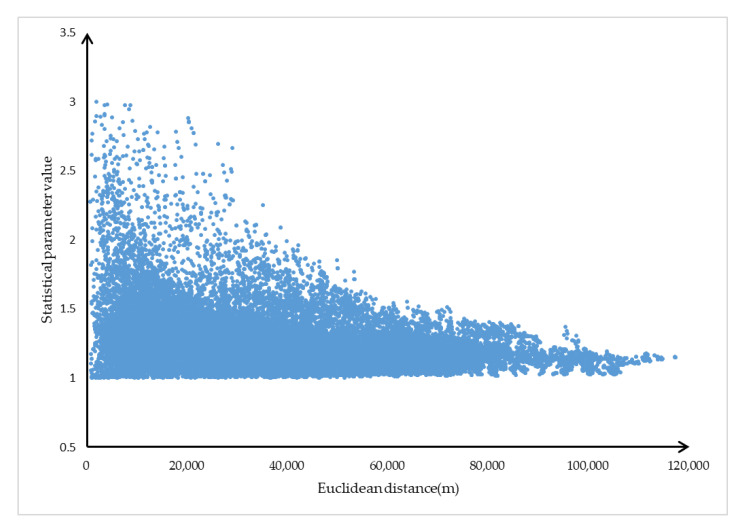
Sample element distribution.

Aiming at the problem of low search efficiency in SPAERSA when the Euclidean distance of the OD pair is large, this paper analyzes *R_OD_* and *E_OD_* data. Take Zhengzhou as an example. First, 400 nodes are systematically extracted from the transportation network of Zhengzhou to construct the set *G_O_*, *G_D_*, so the *G_OD_* includes 40,000 sample elements. We solve $F_{OD}^{i}$, $E_{OD}^{i}$ and $R_{OD}^{i}$ separately for each element in *G_OD_*. If the aggregate *R_OD_* is counted, we can get that the value of the statistical parameter *f* is 1.611 at the 95% confidence level. Figure 1 shows the numerical distribution of *R_OD_* and *E_OD_*. 

It can be seen from Figure 1 that the linear distance between the statistical parameter *f* and the OD pair has a certain functional relationship. The smaller the Euclidean distance in the OD pair, the greater the probability that *f* will get a larger value. The greater the value of Euclidean distance in the OD pair, the greater the probability that the value of *f* tends to 1. Based on this phenomenon, we propose a new calculation method for statistical parameters.

**Figure 2:**
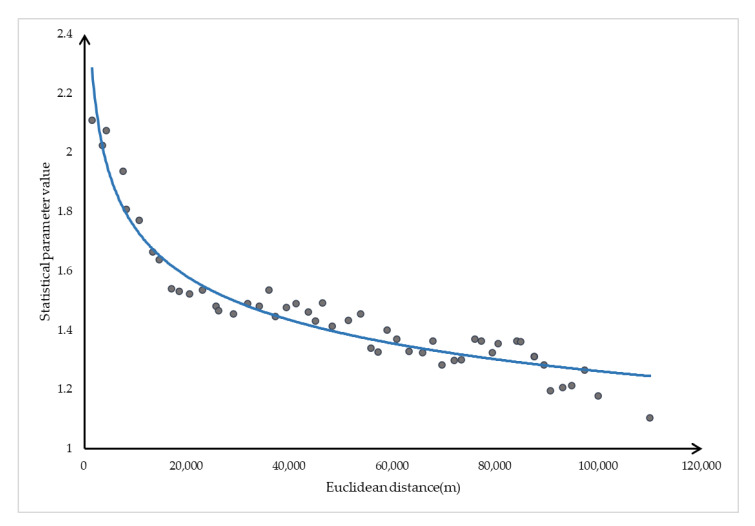
Curve fitting of sample elements in SE.

Assuming that the statistical parameter function *R* satisfies the 95% confidence level, the specific calculation method is:

First, the 40,000 sample elements are sorted according to the Euclidean distance from small to large. We set the Euclidean distance as a group every 2 kilometers and assume that there are C_i_ elements in the i-th group. Then we sort the *f* values in each group from large to small and select the *C_i_* × 5% number in each group to form a new set SE. Finally, we perform curve fitting on the sample elements in the new set. We perform curve fitting on the sample elements in SE to obtain the curve Equation (3) in Figure 2.

*F* = −2.065 × *x*^(0.06163)^ + 5.406 (3)

It can be seen from Figure 2 that the f value of the OD pair decreases with the increase of the Euclidean distance, which avoids the generation of a large number of redundant nodes in the process of traversing the nodes in the search area when the OD pair Euclidean distance is large. The statistical parameter function effectively solves the problem of low search efficiency of SPAERSA when the Euclidean distance of the OD pair is large. However, there are sample points above the curve, which means that a part of the points above the curve cannot obtain the complete solution *S* = (*s*_1_, *s*_2_, *…*, *s_n_*) of the optimal path in the search area.

#### 2.2.3. The Shortest Path Algorithm Based on the Dynamic Ellipse Limit Search Area of the Virtual Boundary

Since the connection direction from the starting point O to the terminal point D basically represents the approximate direction of the shortest path when we plan the path for a given OD pair in the actual urban road network. This means that the final shortest path is basically on both sides of the two nodes, and usually near them. Therefore, OD pairs can get most of the solutions of the optimal path when the complete solution of the optimal path cannot be obtained in the appropriate search area.

Based on the above ideas, this paper proposes a shortest path algorithm based on virtual boundary in dynamic ellipse restricted search area, and the specific process is shown in Algorithm 2. Ω is the set describing the position and state of each edge and the restricted search area. When the start and end points of the edge are in the restricted search area, then Ω = 1. When the start or end of the edge is within the ellipse, then Ω = 2, when the start and end points of the side are outside the ellipse, then Ω = 3. Ψ is the set that restricts the intersection of the search area boundary and the edge in the graph G. α is the Euclidean distance between two points. β is the Manhattan distance between two points. γ is a statistical parameter. $E_{i}$ represents an edge in graph *G*. *G′* represents the edge in the restricted search area.

The Algorithm 2 first constructs a virtual boundary as a virtual solution *VS* = (*vs_i_*, …, *vs_j_*) to replace the solution outside the ellipse area to obtain a virtual complete solution *S′* = (*s*_1_, *s*_2_, …, *vs_i_*, …, *vs_j_*, …, *s_n_*) in the ellipse area when the OD pair cannot obtain the complete solution *S* of the optimal path in the ellipse search area. Then find the optimal complete solution *OS* = (*s_i_*, …, *s_j_*) outside the ellipse for each continuous virtual solution according to the starting point and ending point. Finally, replace the virtual solution in *S′* with the solution in *OS* to get the complete solution *S* = (*s*_1_, *s*_2_, …, *s_i_*, …, *s_j_*, …, *s_n_*). This algorithm can effectively solve the problem that some OD pairs cannot get the optimal path solution due to the small *f* value. The weight value $E_{i}^{Length}$ of the virtual edge is divided into different assignment methods according to the value of $E_{i}^{ID}$ in adjacent nodes. If the $E_{i}^{ID}$ of two adjacent nodes are the same, the value of $E_{i}^{Length}$ is the Euclidean distance between the two points. Otherwise, the value of $E_{i}^{Length}$ is the product of the Manhattan distance between the two points and the statistical parameter.
**Algorithm 2** The Shortest Path Algorithm based on Virtual Boundary in Dynamic Ellipse Restricted Area: *FindPathSPAVBDERA*(*G*, *O*, *D*)**Input:**Graph *G*, origin O, destination D.**Output:**The shortest path from O to D.1:γ ← −2.065 × **sqrt** (α*_OD_*, 0.06163) + 5.406;2:Set O and D as the focal points, α*_OD_* as the focal length, and γα*_OD_* as the major axis length to establish the ellipse equation *P*;3:**while**$\exists E_{i}\in$*G***and**$E_{i}$*. traverse* = false **do**4:**if**$E_{i}.origin\in$*P***and**$E_{i}.destination\in$*P***then**5:Ω← 1, *G_i_′* ← $E_{i}$, $E_{i}$. traverse = true;6: **else if**$E_{i}.origin\;\notin$*P* and $E_{i}.destination\notin$
*P*
**then**7:Ω ← 2, $E_{i}$. traverse = true;8: **else if**$E_{i}.origin\;\in$*P* and $E_{i}.destination\notin \;$*P*
**then**9:Ω ← 3, Ψ ← $E_{i},$
*G_i_′* ← $E_{i}$, $E_{i}$. traverse = true;10:**else**11:Ω ← 4, Ψ ← $E_{i},$
*G_i_′* ← $E_{i}$, $E_{i}$. traverse = true;12:Take the midpoint of the OD pair as the origin, and arrange the elements in Ψ counterclockwise;13:**while**$\exists E_{i}\in$ Ψ and $E_{i}$. traverse = true **do**14:Connect the nodes of the edges away from the center of the ellipse in order;15: **if** Ω = 3 **then**16:$E_{i}^{Length}$← $\alpha_{E_{i}E_{i+1}}$, $E_{i}^{Direction}\;\leftarrow$ 3;17:**else**18: $E_{i}^{Length}$← $\beta_{E_{i}E_{i+1}}$, $E_{i}^{Direction}\;\leftarrow$ 3;19:*S′* ← the shortest path in *G′*;20:vs. ← Set of *n* consecutive virtual solutions *VS_n_* in *S′*;21:**if** vs. = null **then**22:**return** S′; 23:**else**24:**while**∃*VS_i_* ∈ vs. **do**25:*Q_i_* ← Find the shortest path solution based on *VS_i_.origin* and *VS_i_.destination* in *G–G′*;26:*S* ← (*S*′ –vs.) $\cup^{}$
*Q*;27:**return***S*;

### 2.3. Algorithm Performance Analysis 

#### 2.3.1. Algorithm Reliability Analysis

Aiming at the problem that the higher confidence level makes the calculated statistical parameter function value too large, which in turn makes the algorithm search area too large. This paper proposes to choose the statistical parameter function derived from the low confidence level combined with SPAVBDERA to improve the shortest path algorithm of the ellipse restricted search area.

First, we calculate the statistical parameter function under different confidence levels. We select the six confidence levels of 95%, 90%, 85%, 80%, 75%, and 70%, and calculate the statistical parameter functions under these six confidence levels, as shown in Table 1.

**Figure 3:**
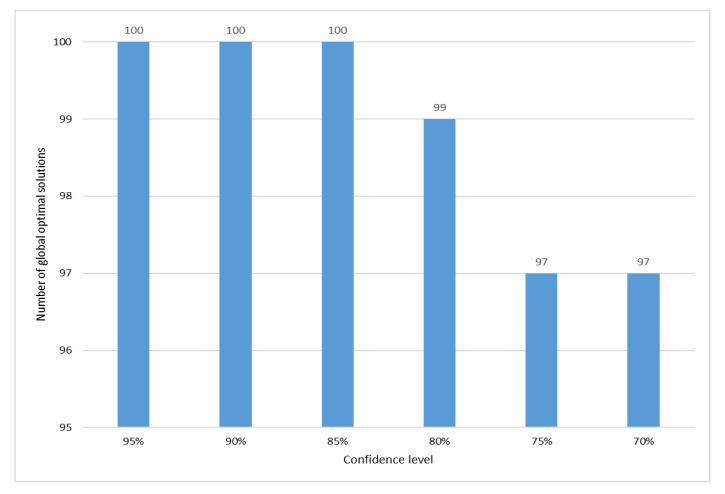
The number of global optimal solutions under different confidence levels in a random sample.

Then 100 sample elements are randomly selected from the road network to calculate the optimal path solution of these 100 sample elements under six confidence levels. Because the Dijkstra algorithm can search for the global optimal solution of the sample elements, the optimal path solution obtained under different confidence levels is compared with the optimal path solution obtained by Dijkstra algorithm to determine whether the optimal path solution obtained under different confidence levels is the global optimal solution, the experimental results are shown in Figure 3.

Finally, the analysis of the sample results shows that SPAVBDERA shows good results at six different confidence levels. Especially SPAVBDERA under the three confidence levels of 95%, 90%, and 85% obtains the global optimal solution in 100 sample elements. Although there are a few sample elements in the remaining three confidence levels that have not obtained the global optimal solution, they have obtained the local optimal solution. It is optimal and reliable to select the 85% confidence level comprehensively considering the accuracy and time complexity of the algorithm.

#### 2.3.2. Algorithm Validity Analysis

**Figure 4:**
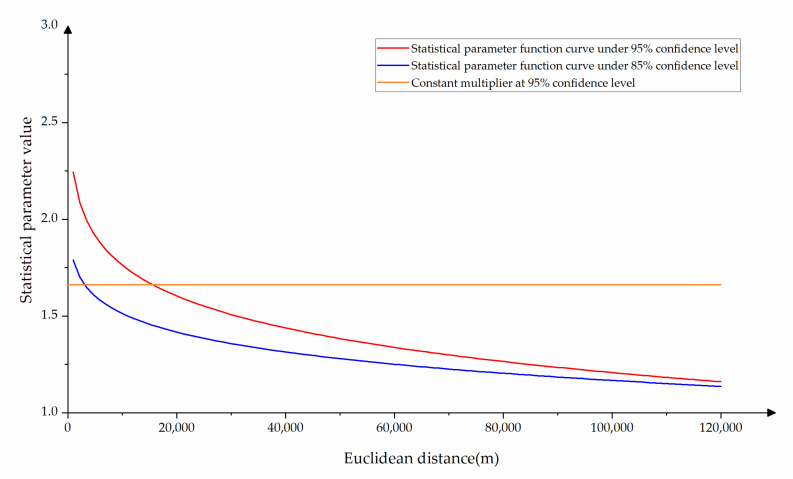
Statistical parameter function graph.

If it is assumed that the nodes in the road network are uniformly distributed in the entire road network plane (that is, the number of nodes is proportional to the area occupied by the area, even if the distribution of local sites is uneven). The actual number of nodes that need to be visited in the search process can be expressed by the area *A* of the searched area, and the time complexity of the search can also be represented as *O* (*A*^2^). Assuming that the time complexity of the shortest path algorithm in the search area restricted by the constant multiplier at the 95% confidence level is expressed as *O* (*A*^2^), the time complexity of SPAVBDERA with the statistical parameter function curve at the 95% confidence level is expressed as $O\;\left(A_{95\%}^{2}\right)$, the time complexity of SPAVBDERA of the statistical parameter function curve at the 85% confidence level is expressed as $O\;\left(A_{85\%}^{2}\right)$. The three statistical parameter function curves are shown in Figure 4.

It can be seen from Figure 4 that the value of the constant multiplier f is 1.611, the value range of the statistical parameter f_95%_ under the 95% confidence level is (1.1603, 2.2451), and the value range of the statistical parameter f_85%_ under the 85% confidence level is (1.1366, 1.7899). Since the time complexity of the algorithm search is proportional to the area of the search area [36], it is easy to get Equation (4).

(4) $$\frac{O\left(A^{2}\right)}{O\left(A_{95\%}^{2}\right)}\;=\;\frac{{\left(\pi \frac{fE_{OD}}{2}\sqrt{{\left(\frac{fE_{OD}}{2}\right)}^{2}-{\left(\frac{E_{OD}}{2}\right)}^{2}}\right)}^{2}}{{\left(\pi \frac{f_{95\%}E_{OD}}{2}\sqrt{{\left(\frac{f_{95\%}E_{OD}}{2}\right)}^{2}-{\left(\frac{E_{OD}}{2}\right)}^{2}}\right)}^{2}}=\frac{f^{4}-f^{2}}{f_{95\%}^{4}-f_{95\%}^{2}}\;\;\;\;\;\;\;\;\;\;\;\;\;\;\;\;\;\;\;\;\;\;\;\;\;\;\;\;$$

It can be seen from Equation (4) and Figure 4 that the time complexity of SPAVBDERA under the 95% confidence level is reduced more than SPAERSA when the Euclidean distance of the OD pair is larger. Similarly, the Equation (5) can be calculated.

(5) $$O\left(A_{95\%}^{2}\right)/O\left(A_{85\%}^{2}\right)=r_{95\%}^{2}\left(r_{95\%}^{2}-1\right)/r_{85\%}^{2}\left(r_{85\%}^{2}-1\right)\;\;\;\;\;\;\;\;\;$$

It can be obtained that the time complexity of SPAVBDERA at 85% confidence level for most OD pairs can be reduced by 21–66% compared to SPAVBDERA at 95% confidence level from Equation (5).

## 3. Results

This chapter mainly compares and evaluates the reliability and effectiveness of the three algorithms (SPAERSA, SPAVBDERA under 95% confidence level and SPAVBDERA under 85% confidence level) by measuring the running time of the algorithm under different Euclidean distances and the number of algorithm traversal nodes on the road network of Zhengzhou. The integrated development environment for running the three algorithms is Microsoft Visual Studio 2017, the development language is C++, the processor is Intel Core i5-4590 @ 3.30GHz quad-core, and the memory is 8GB, the computer operating system is Windows 10, and the database is PostgreSQL 10.0.

**Figure 5:**
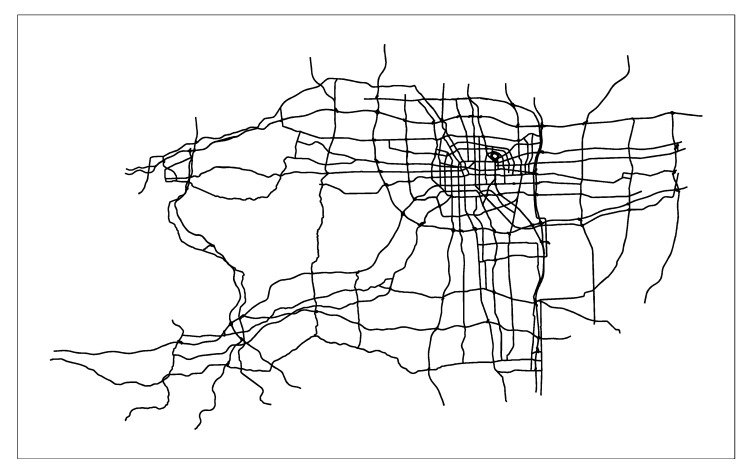
Zhengzhou road network.

**Figure 6:**
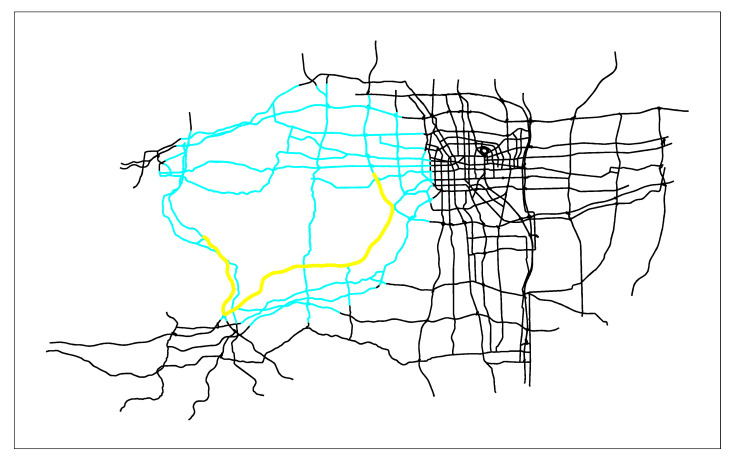
Search results of the shortest path algorithm in the traditional ellipse restricted search area.

**Figure 7:**
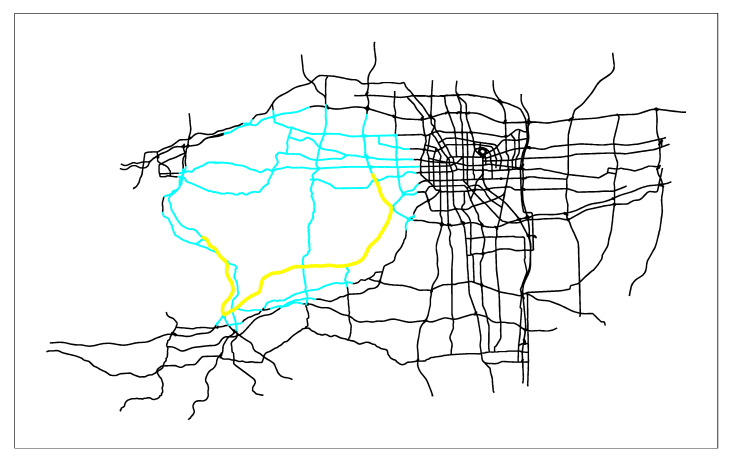
The search results of the shortest path algorithm based on the dynamic ellipse limit search area of the virtual boundary under the 95% confidence level.

**Figure 8:**
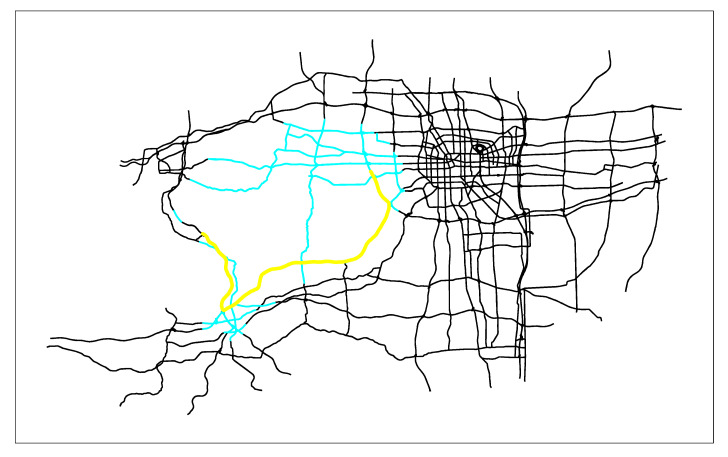
The search results of the shortest path algorithm based on the dynamic ellipse limit search area of the virtual boundary under the 85% confidence level.

First, we combine the actual application of the path planning algorithm in the real-time vehicle navigation system, and apply the three algorithms to the vectorized Zhengzhou road network (the entire road network has 26,206 edges), as shown in Figure 5. Then we solve the shortest path given different starting points and ending points. Due to space limitations, only the starting node 3427 and the terminal node 1057 are given here. Finally, the search results of the three algorithms are obtained. Figure 6 are the search results of SPAERSA, Figure 7 are the search results of SPAVBDERA under the 95% confidence level, and Figure 8 are the search results of SPAVBDERA under the 85% confidence level. The black lines in the three figures indicate the edges of the road network, the blue highlight lines indicate the edges visited by the corresponding algorithm in the process of searching for the shortest path, and the yellow highlight lines indicate the shortest path finally searched results by the corresponding algorithm.

Comparing Figure 6, Figure 7 and Figure 8, it can be seen that the search area scanned by the traditional SPAERSA is an ellipse with the starting node 3424 to the terminal node 1057 as the focal points and 1.611 times the Euclidean distance between the two points as the major axis to obtain the shortest path solution from the starting node 3427 to the terminal node 1057. Although the search area scanned by SPAVBDERA under the 95% confidence level and the 85% confidence level is still an ellipse, the area of the ellipse under these two confidence levels is significantly smaller than the search area scanned by the traditional SPAERSA. After calculation, the search area scanned by SPAVBDERA at the 85% confidence level is an ellipse with the starting node 3424 and the terminal node 1057 as the focal points and 1.324 times the Euclidean distance between the two points as the major axis.

**Figure 9:**
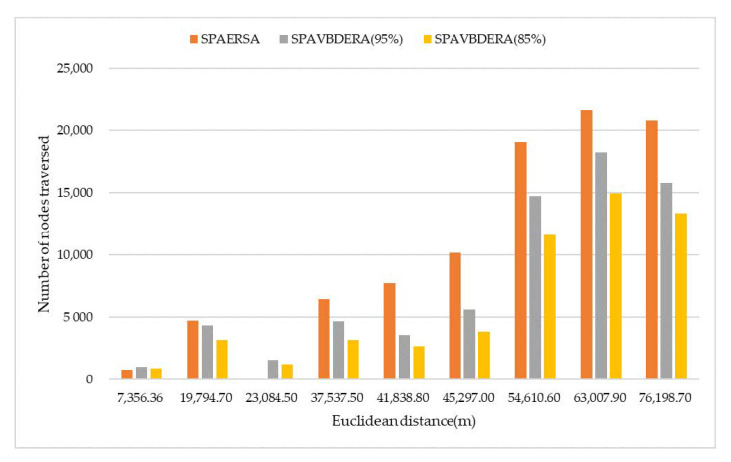
The number of nodes traversed by the three algorithms under different Euclidean distances.

**Figure 10:**
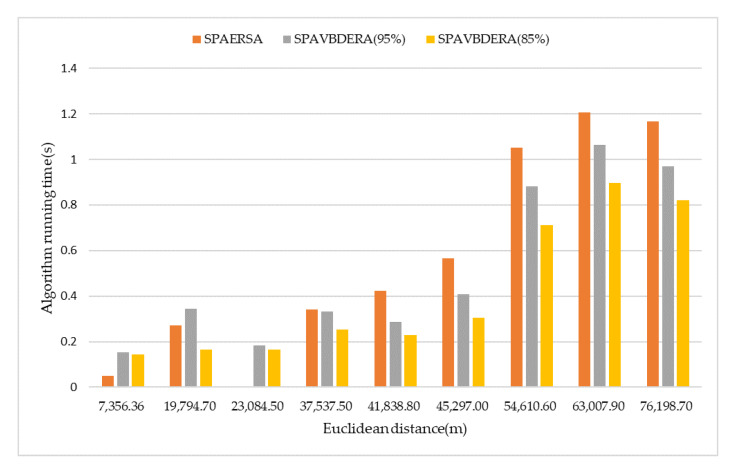
Three algorithms running time under different Euclidean distance.

It can be seen from Figure 6 that the search area scanned by the traditional SPAERSA is able to find the solution of the shortest path, which means that SPAERSA with a lower confidence level cannot search the solution of the shortest path in the search area. it can be seen from Figure 8; however, SPAVBDERA still obtains the shortest path solution under the 85% confidence level. The reason for this phenomenon is that SPAVBDERA obtains the virtual shortest path solution by replacing the solution outside the search area with a virtual solution, then searches for the shortest path solution in a small area. This is basically consistent with the previous theoretical analysis of the algorithm. The performance comparisons of the search results of the three algorithms are shown in Table 2, Figure 9 and Figure 10, respectively.

Table 2 lists the shortest path solutions of the three restricted search area algorithms and Dijkstra algorithm under nine groups of OD pairs with different Euclidean distance, where $S_{Dij}$,$\;S_{SPA}$,$\;S_{95\%}$, and $S_{85\%}$ are represent the shortest path lengths obtained by the Dijkstra algorithm, SPAERSA, and SPAVBDERA, respectively, at 95% confidence level whereas the SPAVBDERA has a confidence level of 85%. In Table 2, the NULL means that the shortest path has not been searched. Figure 9 shows the number of nodes accessed by the three restricted search area algorithms under 9 sets of OD pairs with different Euclidean distances, excluding the number of repeated visits; Figure 10 shows the search time used by the three restricted search area algorithms under nine sets of OD pairs with different Euclidean distances.

From Table 2, the optimal path solution obtained by SPAVBDERA proposed in this paper is always reliable, because the shortest path obtained by SPAVBDERA and Dijkstra algorithm is always consistent. The third set of the data shows that the shortest path solution cannot be obtained in some cases under SPAERSA. This is because of the small statistical parameters of SPAERSA at the 95% confidence level. Therefore, theoretically, the probability when the shortest path solution cannot be obtained is less than 5%, which is a small probability event. It is generally considered that the 5% can be ignored [37].

It can be seen that the number of nodes visited by the algorithm and the running time of the algorithm are directly related to the OD to the Euclidean distance from Figure 9 and Figure 10. As can be predicted, the number of nodes visited by the algorithm and the running time of the algorithm both increase as the Euclidean distance of the OD pair increases. This is because that as the Euclidean distance of the OD pair increases, the search range of the algorithm increases, which increases the number of nodes visited by the algorithm as well as the running time of the algorithm.

Because the statistical parameters of SPAERSA are lower than those of SPAVBDERA under the two confidence levels when the Euclidean distance of the OD pair is small, the search time and the number of search nodes of SPAERSA are lower than that of SPAVBDERA. When the Euclidean distance of the OD pair is larger; however, SPAVBDERA under the two confidence levels shows better performance than SPAERSA.

It can be seen from the nine sets of the experiments that SPAVBDERA under the 85% confidence level is better than under the 95% confidence level in the number of nodes searched and the search time under the premise of ensuring the shortest path solution. Therefore, the application of SPAVBDERA under the 85% confidence level is reliable in this road network.

## 4. Discussion

With the continuous increase of car ownership, more and more vehicles use navigation systems to select routes. The shortest path algorithm is one of the most basic algorithms of the navigation system; therefore, the optimization of the shortest path algorithm is increasingly important.

Accordingly, we proposed a shortest path algorithm based on virtual boundary in dynamic ellipse restricted search to improve the traditional shortest path algorithm in restricted search area. Firstly, we proposed a new calculation method of statistical parameter function based on a more realistic unidirectional road network model. Then, we compared the statistical parameter function at different confidence levels and found that the statistical parameter function at the 85% confidence level is optimal. Consequently we constructed a virtual boundary based on the statistical parameter function under the 85% confidence level, so as to achieve the shortest path solution between two points.

We compared the two groups of SPAVBDERA under different confidence levels and SPAERSA with real road network in Zhengzhou. The experimental results show that under the premise of ensuring the shortest path solution, SPAVBDERA can better adapt to the actual road network. In addition, when the Euclidean distance of the OD pair is slightly larger, the number of search nodes and the search time of the algorithm proposed by SPAVBDERA under the 95% confidence level are significantly reduced compared with SPAERSA, and the reliability of the algorithm is significantly improved as well. Then, we further compared SPAVBDERA under different confidence levels. The experimental results show that the selected SPAVBDERA under the 85% confidence level can theoretically reduce the time consumption by 21–66% compared to the SPAVBDERA under the 95% confidence level.

In the future work, more experiments will be conducted. The necessary traffic control lights in the road network, traffic flow and other factors will be considered as well for in-depth path planning research, realizing the load balance of the road network, which can be better applied to the actual navigation.

## Figures and Tables

**Table 1:** Statistical parameter function under different confidence levels.

Confidence Level	Statistical Parameter Function
95%	*F* = −2.065 × *x*^(0.06163)^ + 5.406
90%	*F* = 4.997 × *x*^(−0.08526)^ − 0.6799
85%	*F* = −0.574 × *x*^(0.09626)^ + 2.906
80%	*F* = −0.08607 × *x*^(0.1943)^ + 1.962
75%	*F* = −0.00278 × *x*^(0.4298)^ + 1.534
70%	*F* = −0.001674 × *x*^(0.4611)^ + 1.471

**Table 2:** Comparison of the shortest path length between Dijkstra algorithm and the three restricted search area algorithms.

Group	$S_{Dij}/m$	$S_{SPA}/m$	$S_{95\%}/m$	$S_{85\%}/m$
1	11,093.9	11,093.9	11,093.9	11,093.9
2	27,898.5	27,898.5	27,898.5	27,898.5
3	50,367.1	NULL	50,367.1	50,367.1
4	75,030.9	75,030.9	75,030.9	75,030.9
5	52,795.6	52,795.6	52,795.6	52,795.6
6	49,771.5	49,771.5	49,771.5	49,771.5
7	84,104.7	84,104.7	84,104.7	84,104.7
8	99,393.8	99,393.8	99,393.8	99,393.8
9	10,0826	10,0826	10,0826	10,0826
